# The Epidemiology of Cancer Among Homeless Adults in Metropolitan Detroit

**DOI:** 10.1093/jncics/pkz006

**Published:** 2019-03-25

**Authors:** Andreana N Holowatyj, Elisabeth I Heath, Lisa M Pappas, Julie J Ruterbusch, David H Gorski, Jeffrey A Triest, Hyo K Park, Jennifer L Beebe-Dimmer, Ann G Schwartz, Michele L Cote, Kendra L Schwartz

## Abstract

**Background:**

Homeless individuals suffer and die disproportionately from chronic diseases and disorders. We describe the epidemiology of cancer among homeless persons in metropolitan Detroit.

**Methods:**

A retrospective cohort study was performed using 1973–2014 data from the Metropolitan Detroit Cancer Surveillance System, a population-based cancer registry and member of the National Institutes of Health-National Cancer Institute’s Surveillance, Epidemiology, and End Results program. Homeless adults were identified through address at diagnosis listed as a homeless shelter, hospital, or supplemental field indicating homelessness. Age-adjusted, sex-specific proportional incidence ratios (PIR) compared cancer incidence proportions by primary tumor site of homeless patients to the nonhomeless referent population. Kaplan-Meier curves depicted unadjusted survival differences in a propensity score matched sample. Differences in 10-year survival were assessed using the score test with a sandwich estimator accounting for matched cluster effects. Statistical tests were two-sided.

**Results:**

A total of 388 individuals experienced homelessness at first primary invasive cancer diagnosis. Statistically significantly higher proportions of respiratory system (PIR = 1.51; 95% confidence interval = 1.28 to 1.79) and female genital system (PIR = 1.83; 95% confidence interval = 1.31 to 2.55) cancers were observed among homeless men and women, respectively. Homeless persons had poorer overall and cancer-reported survival compared with a propensity score matched referent population (median: overall survival, 20.0 vs 38.0 months, respectively, *P* < .001; cancer-reported survival, 38.0 vs 64.0 months, respectively, *P*  < .001).

**Conclusion:**

Disparities in disease burden exist between adults who are experiencing homelessness compared with the nonhomeless population at cancer diagnosis. These findings provide clinically relevant information to understand the cancer burden in this medically underserved population and suggest an urgent need to develop cancer prevention and intervention programs to reduce disparities and improve the health of homeless persons.

From its emergence as a national epidemic in the 1970s, homelessness has afflicted communities all across the United States (1–4). It is estimated that 500 000 to 3 million individuals are homeless in this country, notwithstanding challenges in capturing accurate counts of this transient population (5,6). Investigations of mortality have noted that cancer ranks as a leading cause of death among homeless individuals in Philadelphia and Boston (2,7,8). The high cancer risk profile in this population has been linked to the lifestyle and behaviors of homeless individuals, including increased alcohol and tobacco use, high-risk sexual behaviors, and chronic infections (1,9–17). Homeless persons also have a disproportionately high prevalence of mental illness, which together with a lack of education about cancer prevention and detection (12,18,19) can present as considerable barriers to use of health-care services (3,20–22). However, the burden of cancer among homeless persons has not yet been examined in the Midwest region of the United States.

Among the largest cities in the Midwest, Detroit has garnered attention for its high rates of poverty—where nearly 4 out of every 10 residents live below the poverty level (23). Recently, Chetty et al. (24) reported that Detroit ranked near the bottom for life expectancy among poor residents. Our study sought to describe the burden of cancer among the population of homeless individuals in metropolitan Detroit to establish the foundation for future studies aimed at implementing programs to improve health among the medically underserved in metropolitan Detroit. To study the cancer burden among homeless persons, we obtained data on diagnoses of invasive cancers among patients who were identified as homeless from the Metropolitan Detroit Cancer Surveillance System (MDCSS), a founding member of the National Cancer Institute’s Surveillance, Epidemiology, and End Results (SEER) program. We conducted a study of the cancer burden among adults who were homeless at first primary invasive cancer diagnosis, as well as proportional incidence and survival compared to nonhomeless adults diagnosed with a first primary invasive cancer, in Wayne, Oakland, and Macomb counties of southeast Michigan.

## Methods

### Study Design and Information Sources

**Figure 1.**
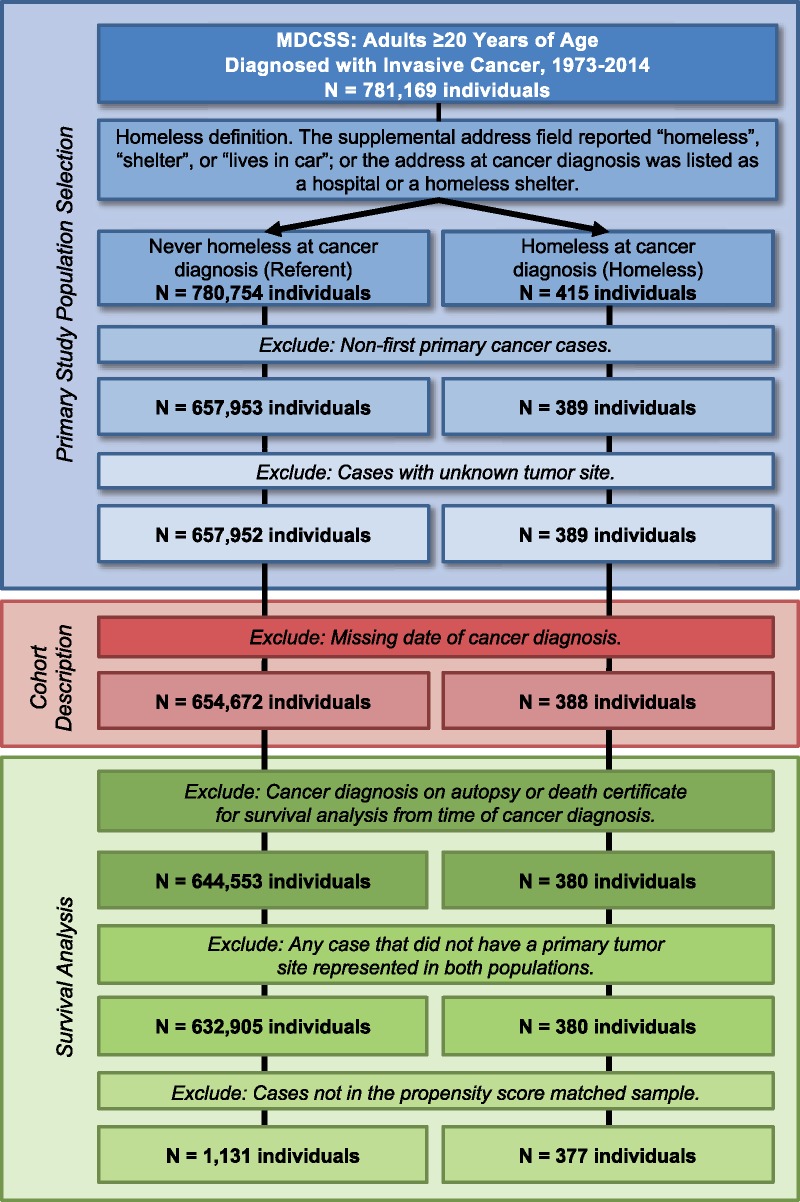
Composition of study population. Cohort description includes clinical and demographic characteristics, cause of death classification, and proportional incidence ratio calculations. MDCSS = Metropolitan Detroit Cancer Surveillance System.

We compared patients who were experiencing homelessness with patients not identified as homeless (referent) at first primary invasive cancer diagnosis in the MDCSS catchment areas of Wayne, Oakland, and Macomb counties of southeast Michigan. The MDCSS is a founding member of the SEER program (25), which has been continuously collecting population-based cancer data since 1973. We obtained deidentified data on 781 169 adults (individuals aged ≥20 years) diagnosed with invasive cancer (regardless of cancer sequence) from 1973 to 2014 (Figure 1). This study was determined as nonhuman participant research by the Institutional Review Board at Wayne State University.

### Identification of Homeless Patients in the MDCSS Population-Based Cancer Registry

The United States Department of Health and Human Services defines a homeless individual as one who lacks housing (without regard as to whether the person is a member of a family), one whose primary residence during the night is a supervised public or private facility (eg, shelter) that provides temporary living accommodations, or an individual who is a resident in transitional housing [42 U.S.C.§254b(h)(2017)]. For this study, individuals experiencing homelessness were identified based on the supplemental address field reporting “homeless,” “shelter,” or “lives in car,” or the address of diagnosis listed as a hospital or a homeless shelter (3). Our cohort was comprised of 388 homeless persons and 654 672 individuals who were not homeless (referent) at diagnosis of first primary invasive cancer (Figure 1).

### Definitions of Cancer Diagnosis and Death

Variables of interest included patient sex, age at diagnosis, race, SEER summary stage, type of reporting source, and year and county of diagnosis. International Classification of Diseases for Oncology site codes were used to define tumor location by primary site and histology according to standard SEER recodes (https://seer.cancer.gov/siterecode/icdo3_dwhoheme/). Cause-of-death classification was based upon the primary cause of death listed on the death certificate (International Classification of Diseases codes).

### Statistical Analysis

All data were analyzed using SAS version 9.4 statistical software (SAS Institute, Cary, NC). All statistical tests were two-sided, with a *P* value less than .05 considered to be statistically significant.

#### 

##### Proportional Incidence Ratios

Age-adjusted, sex-specific proportional incidence ratios (PIRs) and 95% confidence intervals were calculated comparing 388 individuals in the homeless population with 654 672 individuals in the referent population (Figure 1) using the following primary tumor sites: oral cavity and pharynx (lip, tongue, salivary gland, floor of mouth, gum and other mouth, nasopharynx, tonsil, oropharynx, hypopharynx, other oral cavity and pharynx); digestive system (esophagus, stomach, small intestine, colon and rectum, liver and intrahepatic bile duct); respiratory system (nose, nasal cavity and middle ear, larynx, lung and bronchus, pleura, trachea, mediastinum and other respiratory organs); bones and joints; soft tissue (including heart); skin (melanoma of the skin and other nonepithelial skin, excluding basal and squamous); breast; female genital system (cervix uteri, corpus and uterus, ovary, vagina, vulva, other female genital organs); male genital system (prostate, testis, penis, other male genital organs); urinary system (urinary bladder, kidney and renal pelvis, ureter, other urinary organs); eye and orbit; brain and other nervous system (brain, cranial nerves, other nervous system); endocrine system (thyroid, other endocrine including thymus); hematopoietic system (lymphoma, myeloma, leukemia); mesothelioma; Kaposi’s sarcoma; and ill-defined sites.

Calculations were sex specific and adjusted for age using the indirect method with six age groups (<44, 45–54, 55–64, 65–74, 75–84, and 85+ years) (26). PIRs greater than 1.00 indicate that there are proportionally more cancers of a given tumor site among individuals who are homeless at first primary cancer diagnosis than among the referent cohort, after accounting for differences in the distribution of age between populations.

##### Propensity Score Matching

To examine differences in overall and cancer-reported survival between cohorts among individuals with 1 or more survival days from time of cancer diagnosis, a matched comparison sample was created from the referent population (Figure 1; Supplementary Table 1, available online). Standardized differences were calculated for matched covariate descriptive variables and frequencies. A value of *d *= 0.10 or greater (10%) indicates a clinically relevant difference between matched homeless and referent cohorts. Using the Greedy propensity score matching algorithm (27) within exact matched primary tumor site, referent population cases were matched 3:1 to homeless persons. Matching variables used to create the propensity score included primary tumor site, patient sex, race, age at cancer diagnosis, year and county of diagnosis, and SEER summary stage. Our matched cohort was comprised of 377 homeless persons and 1131 individuals from the referent group (Supplementary Table 1, available online).

##### Overall and Cancer-Reported Survival

Survival time was calculated from the diagnosis date to death or date of last follow-up, with follow-up current within 22 months of July 2016. Mean follow-up time was 181 months (SD = 6.2 months), calculated using reverse Kaplan-Meier analysis (28,29). Kaplan-Meier curves were used to evaluate 10-year overall and cancer-reported (cancer as known cause of death) survival using the propensity score matched cohort. The median survival of patients (in months) was calculated based on Kaplan-Meier analysis. Among matched subjects with 1 or more days of survival time, Cox proportional regression analysis assessed differences in survival time groups between patients who were homeless at cancer diagnosis vs matched cancer cases in the referent population. The computation of the robust Sandwich variance estimate aggregates over the cluster, matched to each homeless subject. The reported *P* value is associated with the score test using the Sandwich estimator.

## Results

A total of 388 individuals over the age of 19 years, identified from the MDCSS over the study period, were experiencing homelessness at first primary cancer diagnosis (Figure 1; Table 1). Homeless men comprised 75.5% of the cohort (293 individuals). By race, 48.7% of all homeless individuals in metropolitan Detroit reported as black and 48.7% as white. Mean age at cancer diagnosis among homeless individuals was 60.3 years and statistically significantly differed by sex, with homeless women diagnosed with a first primary cancer at an older age vs homeless men (66.8 vs 58.2 years, respectively, *P* < .0001). Together, 31.4% of all cancer cases in the homeless adult population were diagnosed among individuals 65 years of age and older (Table 1). Nearly one-third (32.0%) of cancer cases among homeless persons were diagnosed at distant tumor stage. Among individuals who were homeless at first primary cancer diagnosis, 39 persons were diagnosed with one or more additional cancers (colon and rectum, lung and bronchus, and esophagus were the most commonly reported tumor sites; data not shown).

**Table 1.** Clinical and demographic characteristics of individuals diagnosed with first primary invasive cancer by the population of individuals who were homeless at diagnosis and the nonhomeless referent population in metropolitan Detroit: MDCSS, 1973–2014

	Homeless	Referent	*P*
Characteristic	No. (%)	No. (%)	
Total	388	654 672	
Sex			<.0001
Male	293 (75.5)	338 676 (51.7)	
Female	95 (24.5)	315 996 (48.3)	
Age at diagnosis, y			<.0001
≤44	34 (8.8)	59 110 (9.0)	
45–54	93 (24.0)	91 658 (14.0)	
55–64	139 (35.8)	158 683 (24.2)	
65–74	66 (17.0)	180 725 (27.6)	
75–84	37 (9.5)	125 354 (19.2)	
85+	19 (4.9)	39 142 (6.0)	
Mean (SD)	60.3 (13.1)	64.3 (14.1)	.04
Race			<.0001
White	189 (48.7)	504 584 (77.1)	
Black	189 (48.7)	140 912 (21.5)	
Unknown	10 (2.6)	9176 (1.4)	
Tumor stage			<.0001
Local	128 (33.0)	283 221 (43.3)	
Regional	74 (19.1)	140 192 (21.4)	
Distant	124 (32.0)	170 614 (26.1)	
Unknown	62 (16.0)	60 645 (9.3)	
Cancer sequence			.06
Only 1 primary	349 (90.0)	567 403 (86.7)	
First of ≥2	39 (10.0)	87 26 (13.3)	
Type of reporting source			.37
Hospital/medical center	377 (97.2)	634 820 (97.0)	
Other	*	9733 (1.5)	
Autopsy/death certificate	*	10 119 (1.6)	
County of diagnosis			<.0001
Oakland	52 (13.4)	177 084 (27.1)	
Macomb	17 (4.4)	124 373 (19.0)	
Wayne	319 (82.2)	353 215 (54.0)	

* Number of cases suppressed due to small cell size. Two-sided *P* value calculations do not include unknown values. MDCSS = Metropolitan Detroit Cancer Surveillance System.

Baseline clinicodemographic characteristics of individuals diagnosed with a first primary cancer in the nonhomeless referent population are also described in Table 1. Clinical and demographic characteristics, including age at diagnosis, sex, race, tumor stage, and county of diagnosis, statistically significantly differed between the homeless and referent population (all *P* < .0001). Compared with the referent population diagnosed with cancer in metropolitan Detroit, individuals experiencing homelessness at first primary cancer diagnosis were more likely to be male, black, and diagnosed at younger ages (Table 1). Individuals who were homeless also presented with later stage disease and were more likely to be diagnosed in Wayne County compared with the nonhomeless referent population. Based on death certificate information and known deaths, 48.7% and 47.4% of individuals among the homeless and referent cohorts, respectively, died from cancer (Table 2). Similar to the referent cohort, homeless individuals had competing causes of death that included diseases of the circulatory or respiratory system (11.3% and 2.8% of individuals in the homeless cohort, respectively).

**Table 2.** Primary causes of death of individuals diagnosed with first primary invasive cancer by the population of individuals who were homeless at diagnosis and the nonhomeless referent population in metropolitan Detroit

	Homeless	Referent
Characteristic	No. (%)	No. (%)
Total	388	654 672
Deaths	301 (77.6)	481 305 (73.5)
Neoplasms	189 (48.7)	310 019 (47.4)
Diseases of the circulatory system	44 (11.3)	91 477 (14.0)
Diseases of the respiratory system	11 (2.8)	21 270 (3.2)
Infectious and parasitic diseases	10 (2.6)	5844 (0.9)
Diseases of the digestive system	10 (2.6)	7956 (1.2)
Diseases of blood and blood-forming organs	—	1235 (0.2)
Complications of pregnancy, childhood, and the puerperium	—	30 (<0.01)
Diseases of the musculoskeletal system and connective tissue	—	747 (0.1)
Congenital anomalies	—	216 (0.03)
Symptoms, signs, and ill-defined conditions	—	1011 (0.2)
Other cause of death*	14 (3.6)	28 493 (4.4)
Unknown cause of death†	23 (5.9)	13 007 (2.0)
Known to be alive	64 (16.5)	158 293 (24.2)
Lost to follow-up‡	23 (5.9)	15 074 (2.3)

* Other cause of death classifications include endocrine, nutritional, and metabolic diseases and immunity disorders; mental disorders; diseases of the nervous system and sense organs; diseases of the genitourinary system; diseases of the skin and subcutaneous tissue; certain conditions originating in the perinatal period; and external causes of death.

† Unknown cause of death includes cases for which no death certificate was available, or a death certificate was available but the underlying cause was not coded.

‡ Follow-up current within 22 months of July 2016.

To identify if proportionally more cancers of a given site were diagnosed among the population of individuals who were homeless at cancer diagnosis than among the nonhomeless referent population, we utilized age-adjusted, sex-specific PIRs by primary tumor site. Cancers of the respiratory system were the most commonly diagnosed first primary malignancies in homeless men (Table 3), with 57.1% of lung and bronchus cancer cases diagnosed at distant stage (48 of 84 men who were homeless at diagnosis of a first primary lung and bronchus cancer). Proportionally, we noted an excess of 51% in incidence of respiratory system malignancies (PIR = 1.51, 95% confidence interval [CI] = 1.28 to 1.79) among homeless men compared with men in the referent population. In particular, homeless men showed an excess of 61% in lung and bronchus cancer incidence compared with men in the referent cancer population (PIR = 1.61, 95% CI = 1.35 to 1.92). In our study, as in the general population, breast cancer was the most commonly diagnosed cancer among homeless women, accounting for 21.1% of all first primary invasive cancer cases (20 of 95 homeless women), but proportional incidence was not higher among homeless women compared with the referent population (PIR = 0.75; 95% CI = 0.52 to 1.08) (Table 3). Cancers of the female genital system (cervix uteri, corpus and uterus, ovary, vagina, vulva, other female genital organs) accounted for over one-quarter (25.6%) of first primary invasive cancers among homeless women (24 cases), with an excess of 83% incidence among homeless women compared with women in the referent cancer population (PIR = 1.83, 95% CI = 1.31 to 2.55). We noted an excess of 193% in the incidence of cervical cancer (PIR = 2.93, 95% CI = 1.51 to 5.67) and an excess of 138% in ovarian cancer incidence (PIR = 2.38, 95% CI = 1.24 to 4.56) among women experiencing homelessness at diagnosis compared with the referent population of women (Table 3).

**Table 3.** Age-adjusted, sex-specific PIR and 95% confidence intervals comparing the proportion of tumors by primary site of men and women who were experiencing homelessness at first primary cancer diagnosis with the nonhomeless referent population diagnosed with a first primary invasive cancer in metropolitan Detroit

	Homeless men	Homeless women
Primary tumor site	No., PIR (95% CI)	No., PIR (95% CI)
Bones and joints	—	≤5*
Brain and other nervous system	≤5*	≤5*
Breast	—	20, 0.75 (0.52 to 1.08)
Digestive system	58, 0.99 (0.79 to 1.24)	21, 1.07 (0.75 to 1.53)
Colon and rectum	27, 0.89 (0.63 to 1.26)	13, 1.09 (0.66 to 1.79)
Other digestive	31, 1.10 (0.79 to 1.53)	8, 1.03 (0.54 to 1.95)
Endocrine system	≤5*	—
Eye and orbit	—	—
Female genital system	—	24, 1.83 (1.31 to 2.55)
Cervix	—	7, 2.93 (1.51 to 5.67)
Ovary	—	8, 2.38 (1.24 to 4.56)
Other female genital	—	9, 1.22 (0.66 to 2.26)
hematopoietic system	19, 0.66 (0.43 to 1.01)	≤5*
Ill-defined sites	8, 1.51 (0.76 to 2.98)	≤5*
Kaposi’s sarcoma	—	—
Male genital system	74, 0.90 (0.74 to 1.09)	—
Mesothelioma	—	—
Oral cavity and pharynx	16, 1.21 (0.76 to 1.94)	≤5*
Respiratory system	91, 1.51 (1.28 to 1.79)	14, 1.11 (0.71 to 1.74)
Lung and bronchus	84, 1.61 (1.35 to 1.92)	14, 1.17 (0.75 to 1.83)
Other respiratory	7, 0.89 (0.43 to 1.84)	—
Skin	≤5*	≤5*
Soft tissue	≤5*	—
Urinary system	14, 0.65 (0.39 to 1.08)	≤5*

* Number of cases suppressed due to small cell size. CI = confidence interval; PIR = proportional incidence ratio.

**Figure 2.**
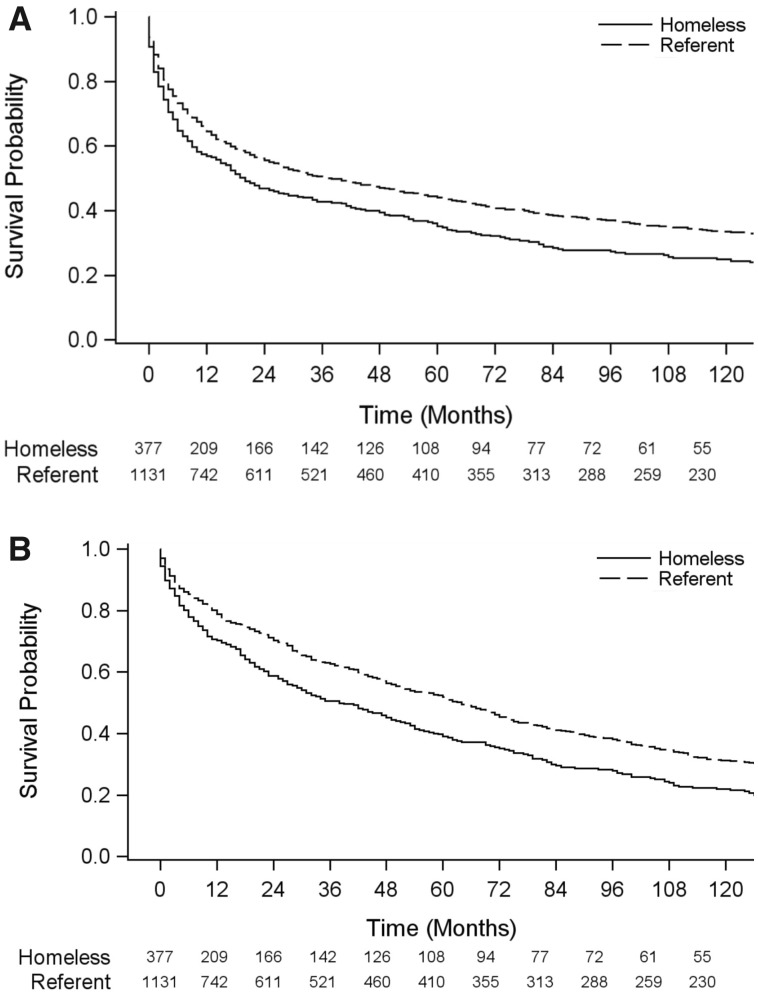
Kaplan-Meier (**A**) overall and (**B**) cancer-reported 10-year survival curves for the propensity score matched cohort of 377 homeless and 1131 nonhomeless referent individuals diagnosed with a first primary invasive cancer in metropolitan Detroit.

To assess disparities in survival between the homeless and nonhomeless referent populations diagnosed with a first primary invasive cancer in metropolitan Detroit, we propensity score matched 377 individuals who were homeless at cancer diagnosis to 1131 individuals from the referent population diagnosed with cancer (Figure 1) by demographic (sex, race, age, year and county of diagnosis) and clinical (primary tumor site, stage) characteristics (Table 1; Supplementary Table 1, available online). In this matched cohort, we observed statistically significantly poorer 10-year survival outcomes for homeless individuals compared with the referent cancer population (*P* < .001) (Figure 2). Nearly 70% (69.8%) of homeless patients were known to have died within 10 years of their cancer diagnosis (263 of 377 individuals). Median overall and cancer-reported survival was statistically significantly lower among homeless patients compared with the referent group (overall survival: 20 vs 38 months, respectively, *P* < .001; cancer-reported survival: 38.0 vs 64.0 months, respectively, *P* < .001).

## Discussion

Our investigation of the burden of cancer among individuals who were experiencing homelessness, as compared with nonhomeless, at diagnosis of first primary invasive cancer in metropolitan Detroit revealed that proportions of cancers of the respiratory system, in particular malignancies of the lung and bronchus, were statistically significantly higher among homeless men when compared with the referent adult population of men diagnosed with cancer. Statistically significantly higher proportions of female genital system cancers, particularly cancers of the cervix uteri and ovary, were observed among homeless women. Together, homeless cancer patients exhibited statistically significantly poorer overall and cancer-reported survival when compared with a propensity score matched referent group. Our study is novel in that it is the first, to our knowledge, to examine the burden of cancer among homeless persons in the Midwest region of the United States, and the first, to our knowledge, to use propensity score matched cohorts to assess the impact of homelessness on cancer outcome.

Across tumor types, early detection of cancer at local disease stage tends to be associated with better prognosis and survival outcomes. In our population of adults who were experiencing homelessness at first primary invasive cancer diagnosis, we found that nearly one-half of cancer cases were diagnosed at distant or unknown tumor stages. Individuals who were homeless at diagnosis of first primary invasive cancer in metropolitan Detroit had statistically significantly lower median survival months and poorer 10-year overall, as well as cancer-reported, survival outcomes compared with propensity score matched cases from the nonhomeless referent population. Previous studies have identified fear, and lack of opportunities and information, as barriers to cancer screening and early detection among homeless persons (12,18,19). In Boston, Baggett et al. (7) observed statistically significantly higher cancer mortality rates among homeless adults compared with general population estimates. In metropolitan Detroit, we noted that disparities in overall and cancer-reported survival between homeless adults and the nonhomeless referent population diagnosed with cancer persisted—even after matching for clinicodemographic characteristics, including tumor stage as a prognostic factor. Together, these findings suggest that other factors (eg, treatment, high cancer risk behaviors, comorbidities) may contribute to this gap in clinical outcomes.

The entire population of homeless individuals is at high risk of death from various competing causes (2,7,8). Although tobacco-related diseases are the most preventable cause of death worldwide (30), studies have demonstrated that smoking-related deaths, including lung cancer, among homeless individuals occur at double the rate compared with those with stable housing (10,14). Indeed, lung cancer accounted for one-quarter of all first primary cancer cases in our homeless cohort. The statistically significant increase in proportional incidence of respiratory system (lung and bronchus) cancers among homeless men may be partly accounted for by the increased uptake of smoking and culture of tobacco use around homeless shelters (9–11,21). In addition, perspectives from individuals experiencing homelessness have included the belief that there is no benefit to worrying about cancer, limited access to resources, and no health insurance (12,18,19,22). These insights may reflect cancer patterns in urban areas of the Midwest region of the United States and emphasize the importance of providing additional resources, including lung cancer screening programs, to aid in the improvement of health among homeless persons.

The proportion of incident female genital system cancer cases was statistically significantly higher among homeless women in Detroit compared with the nonhomeless referent population of women diagnosed with cancer. Previous studies have demonstrated that homeless women tend to engage in more high-risk sexual behaviors and have more sexual partners than stably housed women, with concomitant exposure to blood-borne and sexually transmitted infections (15,16,20). The higher proportion of cervical cancers among homeless women can potentially be attributed to known risk factors for cervical cancer, including limited access and education for screening, persistent infection with human papillomavirus, increased numbers of sexual partners, and smoking (31–36). Although homeless persons have a disproportionately high prevalence of alcohol and tobacco use, high-risk sexual behaviors, mental illness, and chronic infections that contribute to a high cancer risk profile (1,9–17), we acknowledge the limitation of our study that the assessment of chronic conditions, environmental exposures (eg, air pollution), comorbidities, and relevant lifestyle information (eg, HIV/AIDS; tobacco, alcohol, and substance abuse) as well as information about chemotherapy regimens and duration of homelessness were unavailable for assessment in the MDCSS.

Although our findings suggest that disparities in the cancer burden exist between patients who are homeless compared with the nonhomeless referent adult population diagnosed with a first primary invasive cancer in metropolitan Detroit, we acknowledge the limitations of our study. Our analyses were conducted using high-quality data from the MDCSS that allowed for pathologically verified cases to be identified and provided patient address information at time of cancer diagnosis. However, there remains a likelihood of the under-ascertainment of cancer cases among homeless individuals in metropolitan Detroit over the study period, which led to small sample sizes across particular primary tumor sites (eg, cancers of bones and joints) due in part to hospital intake record requirements for a complete address field. Self-reporting of homeless patient addresses could also have included last place of residence or known family address and do not account for duration of homelessness. However, the concordance of our findings with prior studies of lung and cervical cancer among homeless individuals in Boston and Scotland (7,37), and the use of patient address information to identify persons experiencing homelessness at first primary cancer diagnosis, death certificate information, and the propensity score cohort for survival analyses, provided us with a matched sample to study the impact of homelessness on cancer diagnosis and outcomes.

In conclusion, disparities in cancer incidence and outcomes exist between patients who are identified at diagnosis as homeless compared with the nonhomeless referent adult population diagnosed with a first primary invasive cancer in metropolitan Detroit. The results from this study provide clinically relevant information to begin understanding the burden of cancer within the high-risk population of homeless individuals in metropolitan Detroit, and may be useful for other urban areas with similar populations and challenges.

## Funding

This work was partially supported by funding from the Komen for the Cure Graduate Training in Disparities Research Program (GTDR14299438) Fellowship to ANH and MLC, the Cancer Biology Graduate Program at the Wayne State University School of Medicine and Barbara Ann Karmanos Cancer Institute to ANH, and National Institutes of Health under Ruth L. Kirschstein National Research Service Award T32 HG008962 from the National Human Genome Research Institute to ANH, for design and conduct of the study and collection, management, analysis, and interpretation of the data. This work was also supported, in part, by: the Epidemiology Research Core and NIH Center Grant P30 CA022453 to the Karmanos Cancer Institute at Wayne State University and Health and Human Services contract HHSN2612013011I for conduct of the study, and by the University of Utah Study Design and Biostatistics Center, with funding in part from the Huntsman Cancer Institute, the National Cancer Institute of the National Institutes of Health under Award Number P30 CA042014, the National Center for Research Resources and the National Center for Advancing Translational Sciences, National Institutes of Health, through Grant 8UL1TR000105 (formerly UL1RR025764).

## Notes

Affiliations of authors: Department of Oncology (ANH, EIH, JJR, DHG, HKP, JLBD, AGS, MLC), Michael and Marian Ilitch Department of Surgery (DHG), Department of Urology (JAT), and Department of Family Medicine and Public Health Sciences (KLS), Wayne State University School of Medicine, Detroit, MI; Barbara Ann Karmanos Cancer Institute, Detroit, MI (ANH, EIH, DHG, JAT, HKP, JLBD, AGS, MLC); Huntsman Cancer Institute, Salt Lake City, UT (ANH, LMP); Department of Population Health Sciences, University of Utah, Salt Lake City, UT (ANH).

The authors declare no potential conflicts of interest with this work.

We acknowledge the Coalition on Temporary Shelter (COTS), Department of Veterans Affairs at the John D. Dingell Medical Center, and the Community Housing Network, Inc for sharing their resources to aid in this study. We also thank these organizations, as well as other local facilities, for their sustained efforts to help individuals experiencing homelessness in our metropolitan Detroit community.

ANH, KLS, MLC, and EIH contributed to the conception and design of the study. All authors participated in the acquisition, analysis and interpretation of data; and drafting and critical revision of the manuscript for important intellectual content. ANH, LMP, and JJR participated in the statistical analysis and in creating figures. ANH, KLS, MLC, AGS, JLB, and EIH obtained funding and provided support and supervision for this study. ANH and KLS had full access to all of the data in the study and take responsibility for the integrity of the data and the accuracy of the data analysis.

This study was determined nonhuman participant research by the Wayne State University Institutional Review Board.

## Supplementary Material

Supplementary Data
