# Supplementary material for: The Epidemiology of Cancer Among Homeless Adults in Metropolitan Detroit
Source: JNCI Cancer Spectr. 2019 Mar 25;3(1):pkz006. doi: 10.1093/jncics/pkz006 (PMC6433093; doi:10.1093/jncics/pkz006)
Supplement: Supplementary Data [file pkz006_supp.pdf]

Supplementary Materials

Supplemental Table 1. Clinical and demographic characteristics and frequencies of the propensity score matched cohort of 377 homeless and 1,131 non-homeless referent individuals diagnosed with a first primary invasive cancer in metropolitan Detroit: Metropolitan Detroit Cancer Surveillance System (MDCSS), 1973-2014.

	Homeless		***Referent***		
Characteristic	No.	%	No.	%	*d**
**Total**	**377**		**1,131**		
**Age at Diagnosis, y**					0.07
Mean (std)	60.3 (12.3)		59.4 (13.0)		
**Year of Diagnosis**					0.01
Mean (std)	1997 (12.3)		1997 (12.2)		
**Sex**					-0.03
Male	284	75.3	866	76.6	
Female	93	24.7	265	23.4	
**Race**					0.00
White	185	49.1	558	49.4	
Black	183	48.5	550	48.6	
Unknown	9	2.4	23	2.0	
**Tumor Stage**					0.09
Local	126	33.4	421	37.2	
Regional	74	19.6	205	18.1	
Distant	124	32.9	349	30.9	
Unknown	53	14.1	156	13.8	
**County of Diagnosis**					0.06
Oakland	49	13.0	155	13.7	
Macomb	17	4.5	63	5.6	
Wayne	311	82.5	913	80.7	
**Primary Tumor Site Group**					0.00
Exact matched	**	-	**	-	

**A value of d≥0.10 (10%) indicates a clinically relevant difference between matched homeless and referent populations. **Number of cases suppressed due to small cell size.*
